# A review of the application of mesenchymal stem cells in the field of hematopoietic stem cell transplantation

**DOI:** 10.1186/s40001-023-01244-x

**Published:** 2023-08-07

**Authors:** Ting Lin, Yunfan Yang, Xinchuan Chen

**Affiliations:** Department of Hematology, Institute of Hematology, West China Hospital, Sichuan University, 37# Guoxue Xiang, Chengdu, 610041 Sichuan People’s Republic of China

**Keywords:** Hematopoietic stem cell transplantation, Mesenchymal stem cell, Graft-versus-host disease, Hematological disease

## Abstract

Hematopoietic stem cell transplantation (HSCT) is an effective treatment for many malignant hematological diseases. Mesenchymal stem cells (MSCs) are nonhematopoietic stem cells with strong self-renewal ability and multidirectional differentiation potential. They have the characteristics of hematopoietic support, immune regulation, tissue repair and regeneration, and homing. Recent studies have shown that HSCT combined with MSC infusion can promote the implantation of hematopoietic stem cells and enhance the reconstruction of hematopoietic function. Researchers have also found that MSCs have good preventive and therapeutic effects on acute and chronic graft-versus-host disease (GVHD), but there is still a lack of validation in large-sample randomized controlled trials. When using MSCs clinically, it is necessary to consider their dose, source, application time, application frequency and other relevant factors, but the specific impact of the above factors on the efficacy of MSCs still needs further clinical trial research. This review introduces the clinical roles of MSCs and summarizes the most recent progress concerning the use of MSCs in the field of HSCT, providing references for the later application of the combination of MSCs and HSCT in hematological diseases.

## Introduction

Hematopoietic stem cell transplantation (HSCT) is currently the only curative method for most hematological malignancies. Engraftment failure and graft-versus-host disease (GVHD) are two important factors affecting the efficacy of transplantation and the long-term survival of recipients. Currently, hormone and immunosuppressive drugs are used as first-line drugs in the treatment of GVHD, with limited clinical efficacy and only remission in some patients. In addition, the removal of T lymphocytes will weaken the antitumor effect of grafts, leading to tumor recurrence. The long-term use of immunosuppressive drugs and hormones often leads to serious side effects.

**Fig. 1:**
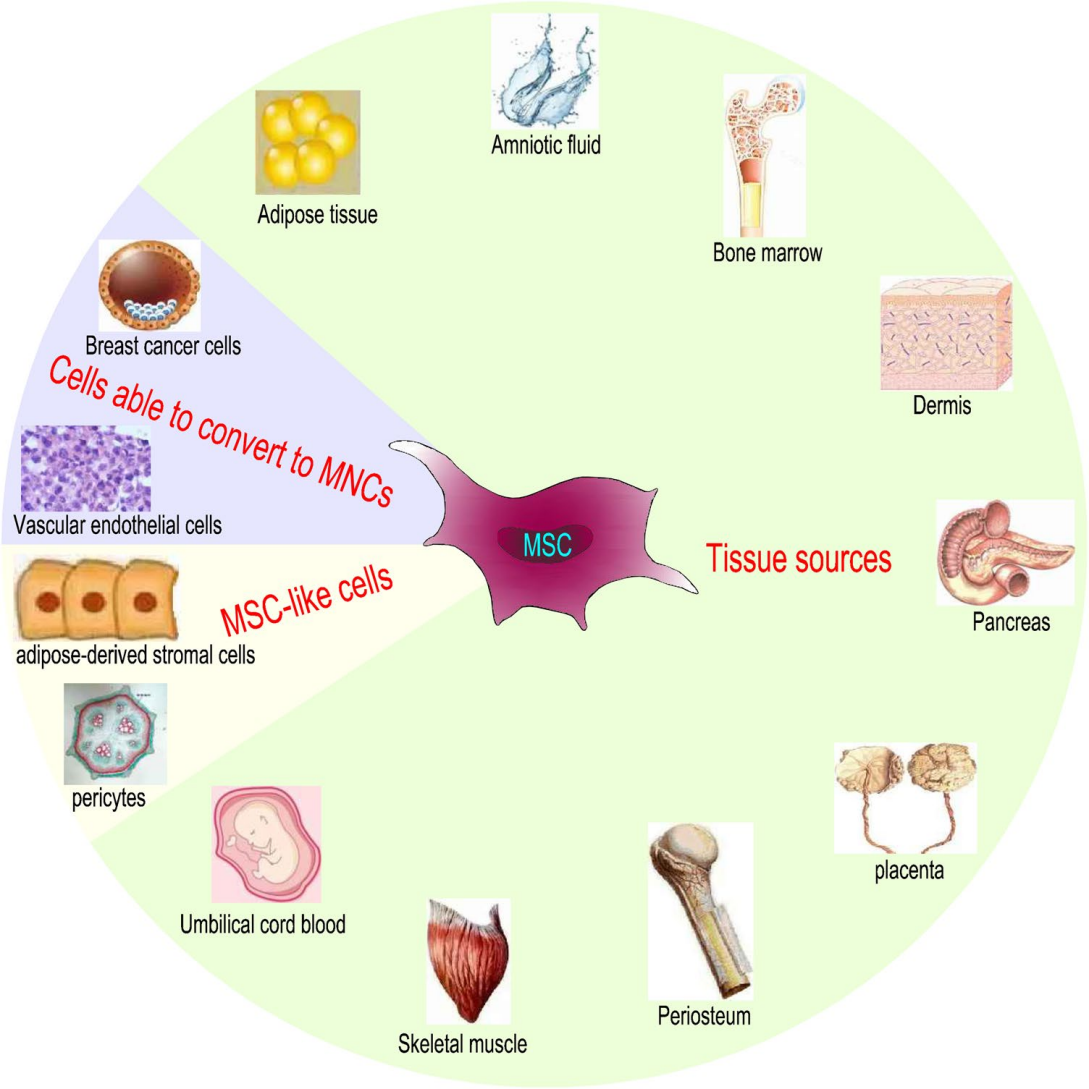
The sources of MSCs are diverse. MSCs can be isolated from tissues, MSC-like cells and cells able to convert to MSCs

Mesenchymal stem cells (MSCs) are stem cells with self-renewal and multidirectional differentiation potential that exist in the bone marrow and are easy to obtain and culture in vitro [1]. The sources of MSCs can vary and can be isolated not only from tissues such as bone marrow, skeletal muscle, periosteum, pancreas, umbilical cord blood (UCB), adipose tissue, amniotic fluid, blood vessels, dermis, and placenta, but also from MSC-like cells and cells able to convert to MSCs (Fig. 1) [2]. Among them, MSCs derived from bone marrow (BM-MSCs) are the most studied, and the bone marrow matrix is the most important source of MSCs. MSCs derived from other sources are also very important. MSCs isolated from UCB have immunogenicity and immunomodulatory effects and have the advantages of simple preparation, easy collection and amplification, no invasive operation and low risk of virus contamination. They can be used as a potential alternative source of BM-MSCs in HSCT [3, 4]. Placenta-derived MSCs (PMSCs) have extensive immunosuppressive effects and can inhibit the proliferation of allogeneic lymphocytes in CD4 and CD8 cell populations [5].

The bone marrow hematopoietic niche is the hematopoietic microenvironment composed of osteoblasts, BM-MSCs, and osteoids, which can accommodate one or more kinds of hematopoietic stem cells (HSCs). BM-MSCs can differentiate into stromal cells such as osteoblasts and fibroblasts and form complex network scaffolds by secreting extracellular matrix, participating in the construction of HSC osteoblasts and the reticular stromal niche, which is an important component of the bone marrow hematopoietic microenvironment. BM-MSCs in the middle regulate the survival, proliferation, differentiation and self-renewal of HSCs by interacting with the adjacent microenvironment (including osteoblasts). BM-MSCs are important components of the hematopoietic niche, not only because they can differentiate into osteoblasts, adipocytes, endothelial cells and other components of the hematopoietic microenvironment, but also because they can regulate the proliferation and differentiation of HSCs [6]. The niche of each tissue affects the characteristics and function of the cell. BM-MSCs play an auxiliary role in the hematopoietic microenvironment, while MSCs derived from UCB (UCB-MSCs) proliferate longer than adult-derived MSCs. Therefore, BM-MSCs showed increased gene expression in the hematopoietic support system, while UCB-MSCs displayed increased genes related to proliferation and cell cycle regulation [7].

**Fig. 2:**
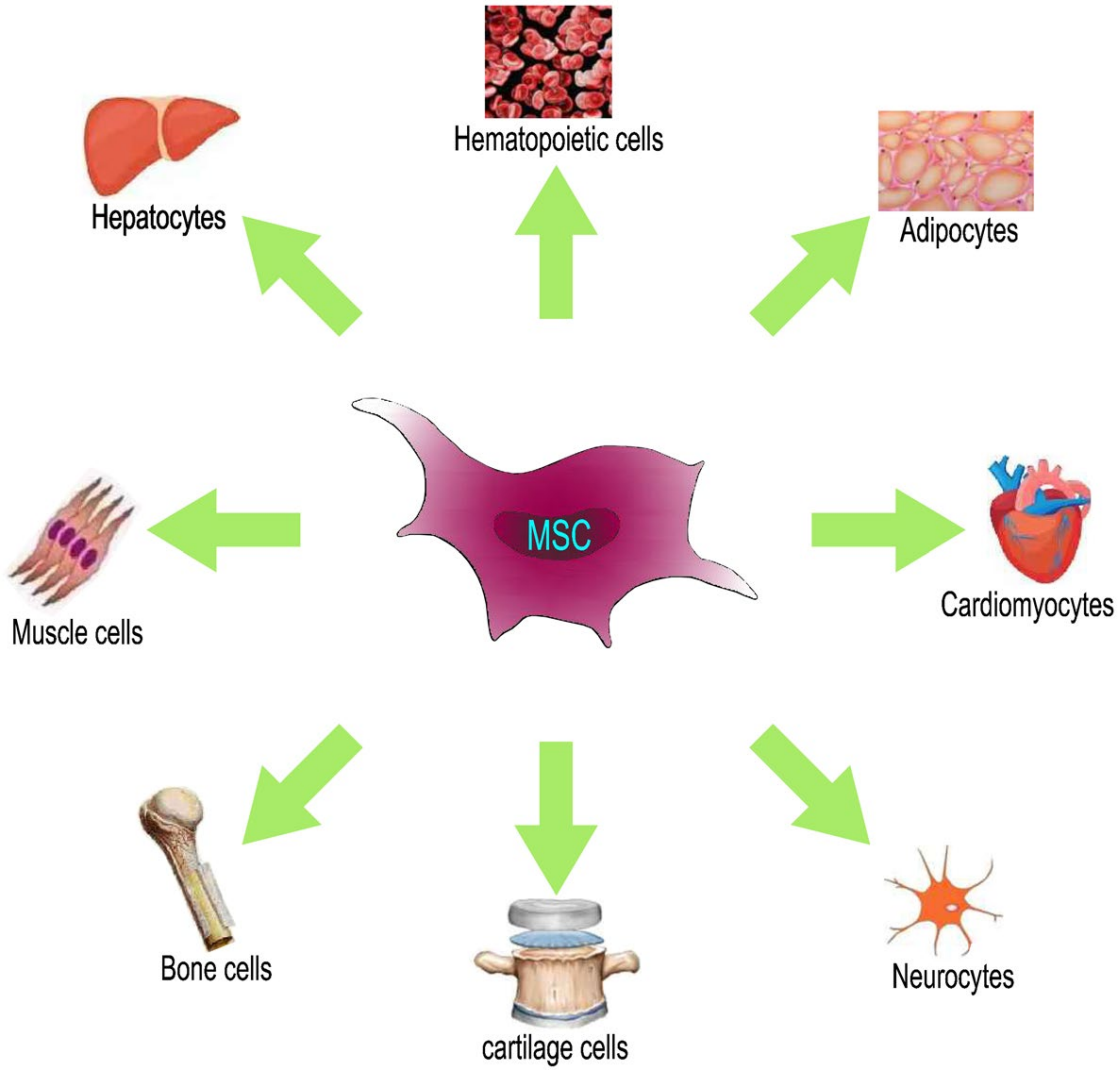
The multidirectional differentiation potential of MSCs. MSCs can differentiate into tissues such as bone, cartilage, and adipose tissue, as well as other mesoderm tissues

MSCs can differentiate into tissues such as bone, cartilage, and adipose tissue, as well as other mesoderm tissues (Fig. 2). The original niche of MSCs can affect their differentiation potential [8, 9]. BM-MSCs, adipose tissue-derived mesenchymal stem cells (AT-MSCs) and UCB-MSCs have a similar high potential for differentiation into osteoblasts and chondrocytes. AT-MSCs have the highest potential for differentiation into adipocytes, followed by BM-MSCs, while UCB-MSCs have difficulty differentiating into adipocytes [10–12]. In addition, MSCs express a variety of surface markers, which also affect the differentiation potential and function of MSCs. CD146-positive PMSCs have stronger osteogenic differentiation potential than CD146-negative PMSCs [13]. When CXCR4 is overexpressed in cells, the ability of MSCs to survive and repair tissue is enhanced after transplantation into damaged tissue [14–16]. Therefore, it is very important to determine the most suitable source of MSCs for different clinical applications.

**Fig. 3:**
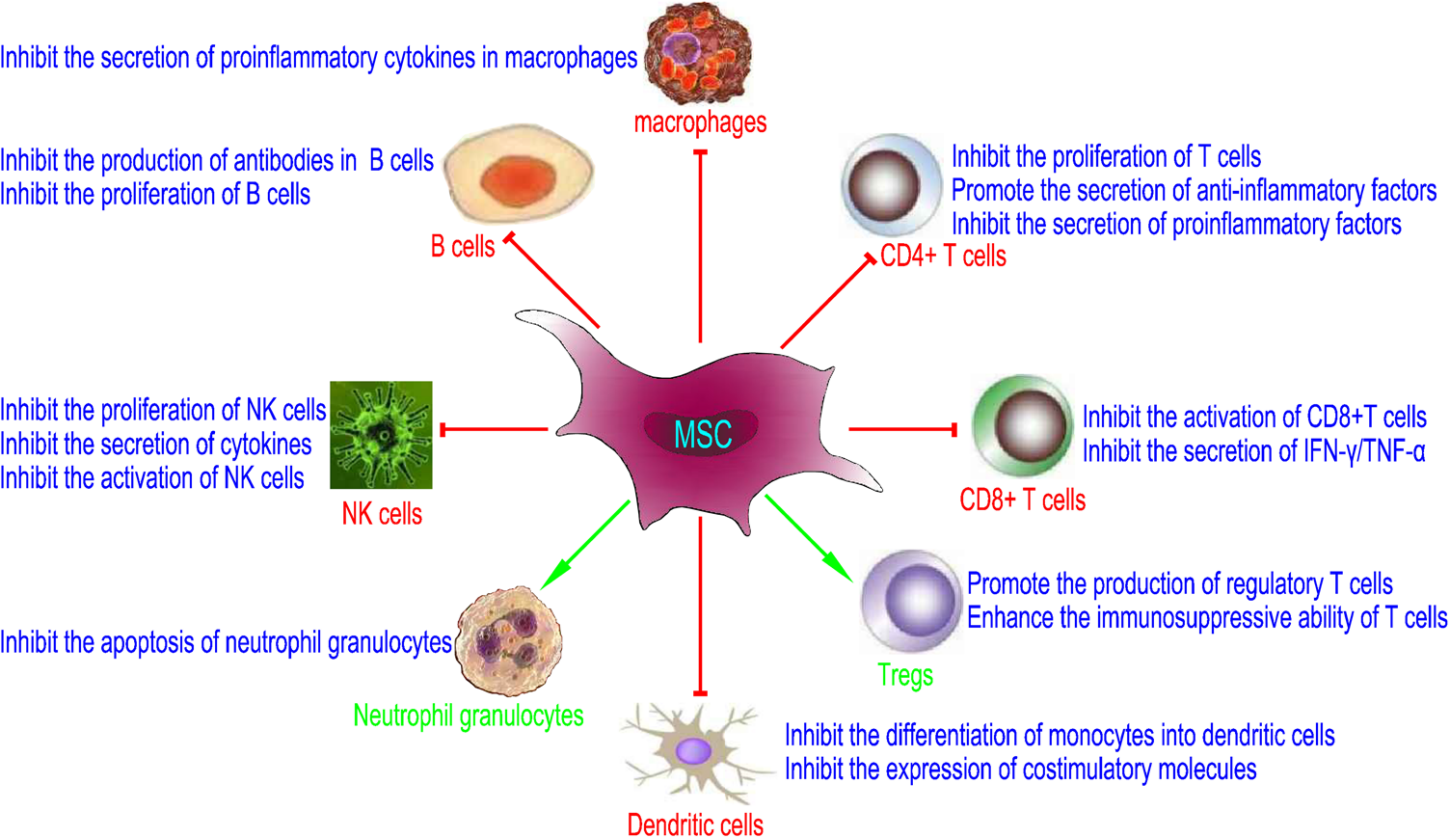
The effects of MSCs on various blood cells

MSCs have strong immunoregulatory activity and can interact with innate and adaptive immune cells in the microenvironment to regulate immune balance [17]. They play an important role in immune regulation through cell‒cell contact, secretion of soluble factors and extracellular vesicles, such as inducing the production of regulatory T lymphocytes, regulating the proliferation, activation and maturation of T and B lymphocytes, and having regulatory functions in both innate and adaptive immune responses [18]. MSCs derived from different tissue origins have different immune functions. BM-MSCs and AT-MSCs could inhibit lymphocyte proliferation more effectively than UC-MSCs, while UC-MSCs and AT-MSCs could induce a higher regulatory T cell /Th17 ratio. There was no significant difference in the prevention and treatment of GVHD among the three types of MSCs [19]. In addition, since MSCs only express HLA-class I antigens and do not express or underexpress HLA-II antigens, they also inhibit the proliferation of T cells. The immunomodulatory functions of MSCs make them ideal for the prevention and treatment of GVHD during allogeneic HSCT (allo-HSCT). The effects of MSCs on various blood cells are shown in Fig. 3.

As shown above, MSCs have the advantages of abundant sources, multidirectional differentiation potential, and easy isolation, culture, and expansion in vitro. These properties make MSCs ideal engineered cells for effective use in cell therapy and gene therapy, and they have been widely used in the field of HSCT [20–22].

In this review, we introduce the latest research on the application of MSCs in the field of HSCT, especially its application in promoting the reconstitution of hematopoietic function, preventing and treating GVHD, and treating transplant-related complications. We also discussed the effect of MSCs on relapse after HSCT in malignant hematological diseases and the application of MSC-derived exosomes in HSCT. Finally, we summarize the problems and prospects for the future development of the application of MSCs in HSCT.

### Promoting the reconstitution of hematopoietic function

Factors such as toxic and side effects of pretransplantation pretreatment and ionizing radiation can seriously damage the bone marrow hematopoietic microenvironment of patients, resulting in delayed hematopoietic reconstitution after transplantation and increased risk of infection and hemorrhage, even engraftment failures [23].

Studies have shown that MSCs, as precursor cells of bone marrow stromal cells, can promote hematopoietic reconstruction by regulating the physiological activity of hematopoietic cells colonized therein. [24]. Lu et al*.* isolated MSCs from full-term human umbilical cord tissue and cultured them in a suitable growth medium. Immunofluorescence and western blotting showed that they expressed mRNA of granulocyte macrophages and granulocyte colony-stimulating factor, as well as a variety of cell growth factors, and had strong hematopoietic support function [25]. Chen et al*.* studied the interaction between BM-MSCs overexpressing CXCR4 and HSCs in mice and confirmed that overexpression of CXCR4 on the surface of MSCs can improve the ability of MSCs to home to bone marrow and spleen, accelerate hematopoietic recovery and promote hematopoietic reconstitution [26]. Studies have also found that BM-MSCs can induce HSCs to home to the bone marrow through the secretion of cytokines such as SDF-1, which is beneficial to restore the normal level of SDF1 in the bone marrow microenvironment, thereby reducing the toxicity of chemotherapy and shortening the recovery time of hematopoietic function [27].

Wang et al*.* reported 35 children with severe aplastic anemia (SAA) who underwent haploid HSCT. They cotransplanted culture-expanded donor-derived BM-MSCs into patients. All children achieved hematopoietic reconstruction and showed continued complete donor chimerism. The median implantation time for myeloid cells was 14 days (range 10–22 days), and the median implantation time for platelets was 18 days (range 9–36 days) [28]. Another study reported 50 patients with refractory/relapsed hematological malignancies who underwent haploidentical HSCT (Haplo-HSCT) combined with infusion of UCB-MSCs, all of whom achieved rapid and stable hematopoietic ability, and the median time for reconstitution of granulocytes and platelets was 12 days and 15 days, respectively [29]. An open-label, randomized phase II clinical study reported that coinfusion of BM-MSCs can promote platelet recovery in patients after haplo-HSCT. The time to platelet concentration > 50 × 10^9^ cells/L was significantly faster in the MSC group than in the non-MSC group, which was 22 days and 28 days, respectively [30].

Infusion of MSCs can also promote hematopoietic recovery in other hematopoietic cell transplantation (HCT), such as umbilical cord blood transplantation (UCBT). Wu et al*.* randomly divided 20 patients with high-risk leukemia into two groups: one group received UCBT and an infusion of UCB-MSCs, and the other group received UCBT alone. Among them, the median time of granulocyte and platelet reconstruction of 8 patients in the UC-MSCs group was 12 days and 30 days, respectively, which was significantly shorter than that of the UCBT alone group (*P* = 0.003, *P* = 0.004), indicating that UC-MSCs can promote hematopoietic function reconstruction and shorten hematopoietic recovery time [31]. Luan et al*.* described a case of SAA that was treated by UCBT. The patient experienced delayed hematopoietic recovery after transplantation, but hematopoietic function improved after the infusion of UC-MSCs. This suggests that early infusion of MSCs after HCT can promote the recovery of hematopoietic function [32]. Wu et al*.* compared 5 patients who received UCB-MSCs in combination with cord blood transplantation (CBT) with 15 patients who received CBT alone. The UCB-MSC group had significantly faster hematopoietic recovery of neutrophils and platelets, the time to achieve neutrophil implantation ranged from 7 to 13 days (median, 11 days), and the time to achieve platelet implantation ranged from 22 to 41 days (median, 32 days) [33].

### Preventing and treating GVHD

GVHD is the main complication of patients receiving allo-HSCT, and it is also an important cause of death, which seriously affects the transplantation effect and often has a poor prognosis. GVHD is mainly caused by T lymphocytes in the donor graft after allogeneic transplantation, which are stimulated by cytokines in the recipient and then attack the alloantigen in the recipient. The main lesions are in the skin, gastrointestinal tract and liver, with a few lesions also occurring in other organs.

GVHD can be divided into acute GVHD (aGVHD) and chronic GVHD (cGVHD) according to the time of onset. aGVHD generally occurs within 100 days after transplantation and mainly manifests as inflammation in the skin, gastrointestinal tract, and liver. It is the most serious complication after allo-HSCT and can be life-threatening. Its incidence is related to the source of grafts, the HLA compatibility of the donor and recipient, and the age of the donor [34]. cGVHD is a clinicopathological syndrome in which donor lymphocytes attack the recipient organs during the process of the immune reconstruction of the donor. It usually occurs 100 days after allo-HSCT and is an important cause of late nonrecurrent death after transplantation. The incidence of cGVHD is approximately 30–70% [35].

At present, the methods for preventing GVHD after allo-HSCT mainly include in vivo and in vitro removal of T lymphocytes and the use of immunosuppressive drugs. However, the removal of T lymphocytes weakens the antitumor effect of grafts and easily leads to tumor recurrence. Long-term use of immunosuppressive drugs and hormones often leads to serious side effects. Therefore, researchers have been working to develop new and efficient GVHD prevention and control strategies. MSCs are expected to be an ideal method for the prevention and treatment of GVHD due to their tissue repair and immunomodulatory functions.

### Prevention of GVHD

Zhao et al*.* found that gingiva-derived MSCs could significantly reduce the infiltration of CD8 + cells and Th1 and Th17 cells while increasing the differentiation of CD4 + Foxp3 + regulatory T cells in lymph nodes [36]. MSCs can inhibit the cytotoxicity of NK cells and have low immunogenicity to T lymphocytes, dendritic cells, B lymphocytes and NK cells [37]. Therefore, they play an important role in regulating autoimmune and inflammatory responses and can effectively prevent the occurrence of GVHD.

It has been reported that infusion of MSCs can reduce the development of aGVHD by 3 times and improve the overall survival of patients after allogeneic HCT compared with standard prophylaxis [38]. Li et al*.* studied whether haplo-HSCT combined with MSC infusion could prevent graft failure and GVHD in 17 patients with SAA. The results showed that the incidence of grade III–IV aGVHD was 23.5%, and the incidence of moderate and severe cGVHD was 14.2%. The average survival time for all patients was 56.5 months, which indicated that haplo-HSCT combined with MSC infusion for the treatment of SAA was safe and could effectively reduce the incidence of severe GVHD and improve the survival rates of patients [39]. Bacigalupo et al. reviewed 375 patients with SAA. They found that infusion of MSCs derived from bone marrow or cord blood during transplantation had a favorable effect on the prevention of aGVHD. The mean rejection rate of these patients was 6%, the incidence of grade II–IV aGVHD was 23%, and the 1 year survival rate was 80% [40]. Gao et al*.* studied the incidence and severity of cGVHD, as well as the changes in T, B and natural killer (NK) cells in patients after repeated infusion of MSCs, and found that the incidence of cGVHD in the MSC group was significantly lower than that in the non-MSC control group. The incidence of cGVHD was 27.4% and 48.4%, respectively, and the 2 year cumulative incidence of cGVHD in the MSC group was lower than that in the non-MSC control group (27.4% vs 49.0%, *P* = 0.021) [41]. The application of MSCs in preventing GVHD has also been reported in other studies (see Table 1).

**Table 1:** Application of MSCs in preventing GVHD

N	Source of MSCs	Diseases	Median dose of MSCs (cells/kg)	Number of infusions	Time between first infusion and HCT	Outcome	Ref.
33	Bone marrow	AML/MDS: 23 ALL: 7 CML: 3	1 × 10^6^	–	27士1.5 days after HCT	Incidence of aGVHD: 9.4% Relapse rate: 25% Graft rejection: 6.2% Death rate: 18.8%	[38]
44	Bone marrow	SAA: 31 VSAA: 13	3.6 × 10^8^	2	Within 6 h before HCT	Incidence of II–IV aGVHD: 29.3% Incidence of cGVHD: 14.6% 1-year OS: 77.3%	[42]
20	Bone marrow	AML: 7 NML:5 MM: 5 Other: 3	–	1	30–120 min before HCT	Incidence of aGVHD: 35% Incidence of cGVHD: 65% 1 year OS: 80% 1 year PFS: 60%	[43]
17	Bone marrow	PMF	6.5 × 10^6^		The day of HCT 7 Days after HCT	Cumulative incidence of aGVHD: 62% Cumulative incidence of overall cGVHD at 2 years: 63% Cumulative incidence of moderate to severe cGVHD at 2 years: 17%	[44] ,
17	Cord blood	SAA	4 × 10^6^	1	6 h before HCT	Incidence of III–IV aGVHD: 23.5% Incidence of cGVHD: 14.2% 3 month survival rates: 88.2% 6 month survival rates: 76.5%	[39]
62	Cord blood	AML: 43 ALL: 14 MDS: 5	–	3.7	–	2 year cumulative incidence of cGVHD: 27.4% 2 year OS: 66.1%	[41]
77	Cord blood	SAA or VSAA: 72 SAA& PNH: 5	5 × 10^5^	1	4 h before HCT	Incidence of II–IV aGVHD: 18% Incidence of III–IV aGVHD: 10% Incidence of extensive cGVHD: 7% 1 year OS: 93.1% 5 year OS: 87.9%	[45]

*MSCs* mesenchymal stem cells, *HCT* hematopoietic cell transplantation, *SAA* severe aplastic anemia, *aGVHD *acute GVHD, *cGVHD* chronic GVHD, *OS* overall survival, *AML* acute myelogenous leukemia, *NML* non-Hodgkin lymphoma, *MM* multiple myeloma, *PFS* progression-free survival, *PMF* primary myelofibrosis, *PNH* paroxysmal nocturnal hemoglobinuria

### Treatment of aGVHD

Corticosteroids are the first-line treatment for aGVHD, with an overall response rate of approximately 67% to 80%. Patients with hormone-resistant aGVHD, even if receiving second-line therapy, will also experience unsatisfactory efficacy due to drug resistance, infection or other factors, and the overall survival rate (OS) is less than 10%. The 5 year expected survival rates for patients with grade III and grade IV aGVHD are 25% and 5%, respectively [46].

At present, BM-MSCs are the most studied and widely used MSCs in the treatment of severe steroid-resistant aGVHD. In a multicenter, phase II experimental study, Frassoni et al*.* reported that 55 patients with severe and steroid-resistant aGVHD were treated with MSCs. The median dose of these cells was 1.4 × 10^6^ (min–max range 0.4–9 × 10^6^) cells per kg bodyweight. Thirty-nine patients responded to treatment with MSCs, and no patients had side effects during or after infusions of MSCs [47]. In a study of MSCs combined with basiliximab, a calcineurin inhibitor, in the treatment of steroid-resistant aGVHD, Zhao et al. found that only 17 of 99 patients did not respond to MSCs, 56 (56.6%) achieved CR and 26 (26.3%) achieved PR, and the OR rate of the MSC group was significantly higher than that of the control group at day 28 [48]. Another phase 3 randomized study conducted by Kebriaei et al*.* showed that patients receiving MSC infusion had a higher durable complete response and a higher overall complete or partial response rate than patients receiving placebo (29% vs. 5%; *P* = 0.047) [49]. Dotoli et al*.* reported that in 46 patients with hormone-resistant grade III–IV aGVHD (78.3% grade IV aGVHD), 23 patients (50%) improved their clinical symptoms after receiving third-party BM-MSC therapy, with 13% complete remission, 60.9% partial remission, and 26.1% short partial remission. The 1 year and 2 year OS were 19.6% and 17.4%, respectively, and the OS of patients who responded to MSCs was significantly higher than that of patients who did not respond [50]. A meta-analysis of 13 studies including 301 patients showed that when MSCs were used for the treatment of steroid-resistant aGVHD, the overall response (OS) rate was 68.1%. Of the 301 patients, 136 patients showed a CR, and 69 patients showed a PR or mixed response (MR). Their results showed that MSCs were more effective in treating patients with grade II aGVHD than those with grade III–IV aGVHD, and for patients with poor initial treatment effect of MSCs, prolonging the treatment cycle can improve the efficacy [51]. Another meta-analysis involving 336 patients reported that when MSCs were used in the treatment of aGVHD, the weighted average of patients surviving at 6 months in gastrointestinal, hepatic, and skin aGVHD were 49%, 28%, and 49%, respectively [52]. Bueren et al*.* demonstrated for the first time that AT-MSCs can effectively control GVHD caused by allo-HSCT by expanding human AT-MSCs and mouse AT-MSCs in vitro [53]. In a randomized controlled trial to investigate the efficacy of MSCs in the treatment and prevention of GVHD, 47 patients with steroid-resistant GVHD were enrolled, of whom 28 patients were treated with MSCs, with a median number of 4 infusions. MSCs were given at a median dose of 1 × 10^6^ cells/kg weekly, and the overall remission rate was 75% in the MSC group compared with 42.1% in the non-MSC group (*P* = 0.023). Meanwhile, the incidence of cGVHD and extensive cGVHD in the MSC group was significantly reduced, and the 2 year cumulative incidence of cGVHD was 31.5% ± 10.1% in the MSC group and 79.2% ± 12.7% in the non-MSC group [54]. A summary of the application of MSCs in treating aGVHD is presented in Table 2.

**Table 2:** Application of MSCs in treating aGVHD

*N*	Source of MSCs	Age of patients (median/range, years)	Diagnosis	Grade of aGvHD	Median dose of MSCs (cells/kg)	Time of MSCs infusion after aGVHD	Outcome	Ref.
75	Bone marrow	8[2–17]	ALL: 18 AML: 16 CML: 1 Other: 40	IV	2 × 10^6^	Day + 72	OR: 46 100 day survival: 35	[55]
25	Bone marrow	33 [5–66]	ALL: 9 AML: 8 MDS: 3 Other: 5	III–IV	2 × 10^6^	Day + 2	CR: 6 PR: 9 MR: 4 NC: 1	[56]
26	Bone marrow	6.5 [1–19]	ALL: 8 AML: 5 MDS: 6 Others: 7	III–IV	1.39 × 10^6^	–	CR: 5 PR: 15 No response: 6	[57]
40	Bone marrow	27.8 [1–65]	-	II–IV	1.5 × 10^6^	< Day + 30 (23) > Day + 3 (15)	1 year OS: 50.0% 2 years OS: 38.6%	[58]
35	Bone marrow	7 [0.7–18]	ALL: 10 AML: 6 MDS: 7 JMML: 5 Other: 7	III–IV	2 × 10^6^	Day + 13	CR: 65% 2.9 years OS: 37%	[59]
25	Bone marrow	< 40 years: 6 40–60 years: 8 > 60 years: 11	AML: 6 MDS: 7 Other: 12	II–IV	1.1 × 10^6^	–	CR: 11 PR: 6 No response: 7	[60]
14	Bone marrow	52 [4–62]	AML: 4 MDS: 3 ALL: 3 Other: 4	II–III	2 × 10^6^	Day + 25	CR: 8 PR: 5 No response: 1	[61]
50	Bone marrow	19 [1–69]	ALL: 10 AML: 15 Other: 25	II–IV	1.05 × 10^6^	Day + 27	3.6 years OS: 56%	[62]
2	Adipose tissue	15 12	ALL:1 AML:1	III IV	1 × 10^6^	Day + 68 Day + 97	CR: 12 months CR: 24 months	[63]
6	Adipose tissue	38 [22–49]	ALL:3 AML:3	III–IV	1 × 10^6^	–	CR: 5 No response: 1	[64]
1	Umbilical cord	4	SAA	IV	3.3 × 10^6^	Day + 13	CR: 18 months	[65]
46	Bone marrow Adipose tissue	–	–	II–IV	1 × 10^6^	–	OR: 58.7% 2 year survival for responders: 51.85%	[66]

*MSCs* mesenchymal stem cells, *aGVHD* acute GVHD, *ALL* acute lymphoblastic leukemia, *AML* acute myelogenous leukemia, *CM*L chronic myeloid leukemia, *OR* overall response, *MDS* myelodysplastic syndrome, *CR* complete response, *PR* partial response, *MR* mixed response, *NC* no change, *OS* overall survival, *JMML* juvenile myelomonocytic leukemia, *SAA* severe aplastic anemia

### Treatment of cGVHD

In recent years, many studies have confirmed that MSCs have a certain effect on the prevention and treatment of cGVHD and will not increase the risk of recurrence and infection. In a multicenter, double-blind, randomized controlled trial, 124 patients with haploid HSCT were divided into an MSC group and a non-MSC control group. Peng et al*.* found that MSCs may play an anti-cGVHD role by promoting the proliferation of CD5 + B cells and promoting their secretion of IL-10. They believe that CD5 + regulatory B cells (CD5 + Breg cells) may be the target cells of MSCs in the treatment of cGVHD [67]. Lim et al*.* found that MSCs can significantly reduce the expression of skin cGVHD in mice through animal experiments. The mechanism is that MSCs can downregulate the expression of CD4 + T lymphocytes CCR4 and CCR8 and monocyte macrophages CCR1, reduce the level of skin CCL1, CCL3 and other chemokines, and ultimately inhibit the migration and infiltration of inflammatory cells into the skin [68]. A clinical study showed that after 16 patients with cGVHD were infused with BM-MSCs or AT-MSCs at a dose of 1 × 10^6^ cells/kg of body weight, 65.50% of the patients achieved an overall response, and the 2 year survival rate for responders was 70%. This suggests that MSC infusion may be a new treatment for cGVHD [66]. A summary of the application of MSCs in treating cGVHD is presented in Table 3.

**Table 3:** Application of MSCs in treating cGVHD

*N*	Source of MSCs	Diseases	Age of patients (median/range, years)	Dose of MSCs (cells/kg)	Number of infusions	Median time of MSCs infusion after cGVHD	Outcome	Ref.
23	Bone marrow	AML: 8 ALL: 4 CML: 6 Other: 5	31 [14–51]	1 × 10^6^	3	–	1 year CR: 16 1 year PR: 4 1 year minor PR: 3	[67]
19	Bone marrow	AML: 6 ALL: 4 CML: 8 MDS: 1	28 [18–39]	0.6 × 10^6^	2	35.6 weeks	CR: 10 PR: 4 2 year OS: 77.7%	[69]
4	Bone marrow	AML: 2 ALL: 1 MM: 1	41 [38–43]	1–2 × 10^7^	4–8	17.3 months	OS: 100%	[70]
14	Adipose tissue	Lymphomas: 9 Acute leukemias: 4 Myeloma: 1	51 [24–60]	1 × 10^6^ (9) 3 × 10^6^ (5)	–	–	CR: 8 PR: 2 1 year OS: 71.4% Median survival: 45.3 weeks	[71]

### Treating transplant-related complications

MSCs also play an important role in the treatment of many complications after HSCT. Ringde´n et al. treated 10 patients with complications after allo-HSCT with MSCs derived from HLA-identical, haploidentical, or third-party sources, including 7 patients with grade II–V hemorrhagic cystitis (HC), 2 patients with pneumomediastinum, and 1 patient with colon perforation and peritonitis. The median dose of MSCs was 1.0 × 10^6^/kg intravenously. After infusion of BM-MSCs, 5 patients with HC were cured, the median time of the disappearance of gross hematuria was 3 days, 2 patients with pneumomediastinum recovered, 1 patient with intestinal nonsteroidal resistant GVHD with perforated diverticulitis and peritonitis recovered to normal, and 2 patients died of multiple organ failure due to reduced blood transfusion times [72]. Wang et al. reported using third-party donor-derived UC-MSCs to treat 7 patients with severe late-onset HC after allo-HSCT, with a median dose of 1.0 (0.8–1.6) × 10^6^/kg. Five patients responded to treatment, and 3 patients experienced rapid disappearance of gross hematuria after 1 infusion of UC-MSCs [73].

It has been suggested that one possible mechanism of primary graft failure is alteration of the mesenchymal interstitial compartment. Atay et al. confirmed that MSCs can provide physical support in the bone marrow niche and secrete soluble factors to control and maintain HSCs. [74]. It has also been suggested that MSCs may reduce the risk of graft failure in haploidentical HSC transplant recipients due to their potent immunosuppressive effect on alloreactive host T lymphocytes that evade the conditioning regimen [75]. Therefore, infusion of MSCs has become a safe and feasible treatment strategy to improve the outcome of graft failure.

Fang et al. reported the use of AT-MSCs for salvage treatment in 2 patients with pure red cell aplasia (PRCA) after ABO-mismatched HSCT. Before treatment, the hemoglobin levels of the two patients were 4.9 g/L and 5.1 g/L, and the reticulocyte percentages were 0.04 and 0.06, respectively. After the infusion of AT-MSCs at a dose of 1.5 × 10^6^/kg of the patients’ weight, the hemoglobin levels were 13 g/L and 12 g/L, and the reticulocyte percentages were 1.1 and 0.9, respectively [76].

Another study reported 3 patients who developed acute respiratory distress syndrome (ARDS) after allogeneic HCT. The first patient had clearance of pulmonary infiltrates after infusion of BM-MSCs but eventually died of multiorgan failure. Another patient who received BM-MSC infusion died of Aspergillus infection. The third patient, who was treated with PMSCs, had a significant therapeutic effect and was still alive after 7 years with a Karnofsky score of 100% [77].

### Application of MSCs in the relapse of hematological malignancies after HSCT

The effect of MSCs on relapse in hematologic malignancies after HSCT is controversial**,** and most studies have shown that patients who receive MSCs have a better prognosis than those who do not [43]. Gao et al. compared the cumulative incidences of relapse in patients after haplo-HSCT who received MSC infusion with those who did not, which were 30.6% and 32.3%, respectively, with no significant difference. In addition, they did not observe any adverse reactions associated with the infusion of MSCs, suggesting that MSC infusion is safe and reliable [41]. Studies have also suggested that MSCs have immunomodulatory effects on NK cells, which can preserve the activity of NK cells, enhance the graft-versus-leukemia effect and reduce the risk of leukemia recurrence [78]. However, a pilot clinical study reported a higher incidence of relapse in patients infused with MSCs than in the control group. A total of 25 patients with hematologic malignancy after transplantation were enrolled in the study and divided into the MSC group (*n* = 10) and the non-MSC group (*n* = 15). The number of recurrent patients was 6 (60.0%) in the MSC group and 3 (20.0%) in the non-MSC group. The 3 year disease-free survival rates were 30.0% for the MSC group and 66.7% for the non-MSC group [79].

### Application of exosomes derived from MSCs

Exosomes are extracellular vesicles (EVs) with a straight diameter of 40–100 nm that are released into the extracellular space after the fusion of intracellular multivesicular bodies with the cell membrane. Studies have shown that the immunosuppressive and anti-inflammatory properties of MSCs are mainly due to the release of EVs [80]. Exosomes derived from MSCs (MSC-Exos) can regulate the balance of Treg/Th17 in AA patients through SphK1-mediated exosome enrichment [81]. Studies have demonstrated that MSCs-Exo can be used to attenuate the activated immune system and relieve symptoms in patients with aGVHD, and the infusion was well tolerated with no side effects reported [82]. Wang et al*.* found that exosomes derived from human UC-MSCs can significantly reduce the frequency and absolute number of CD3^+^ CD8^+^ T cells, reduce the levels of IL-2, TNF-α and IFN-γ in serum, and increase the level of IL-10 in serum in an allo-HSCT mouse model, thus preventing life-threatening aGVHD by regulating immune responses [83]. Lai et al*.* found that MSC-Exos can prolong the survival of cGVHD mice and improve their pathological damage through many mechanisms, including inhibiting the migration and infiltration of CD4 + T cells into the lung, reducing the proportion of Th17 cells in the spleen and lymph nodes, inducing Treg cells, and reducing the release of inflammatory factors such as IL-17A, IL-21, and IL-2 [84]. It has also been reported that MSC-Exos can alleviate the symptoms of scleroderma cGVHD mice by inhibiting the activation of skin macrophages, B-cell immune response, and production of TGF-β and smad2 in the skin [85].

### Application of placenta-derived decidua stromal cells (DSCs)

With the increasing application of MSCs in regenerative medicine, it has been found that placenta-derived DSCs have stronger immunosuppressive effects than MSCs. They can induce FOXP3( +) regulatory T cells, promote the anti-inflammatory cytokine profile and suppress alloreactivity in a contact-dependent manner, which is expected to be a promising method for the treatment of severe aGVHD [86]. Ringden et al. studied the safety and efficacy of placenta-derived DSCs in the treatment of severe aGVHD. The results indicated that DSCs were safe and that patients treated with DSCs had much better survival than those treated with conventional immunosuppressive therapy [87]. In another study reported by Baygan et al. they evaluated the side effects and safety of using DSCs in 34 patients with aGVHD and 10 patients with HC, with a median cell dose of 1.5 (range 0.9–2.9) × 10^6^ DSCs/kg. They also compared the therapeutic effects of DSCs with those of 30 aGVHD patients and 10 HC patients who were not treated with DSCs. Only 3 patients had transient reactions during DSC infusion. The 1 year survival rate of aGVHD patients in the DSC infusion group was 67%, and that of HC patients was 90%, which was significantly higher than that of the control group. Therefore, infusion of DSCs is safe and effective without significant side effects [88].

## Conclusions

In summary, MSCs, as a kind of nonhematopoietic stem cell with multiple differentiation potential, have shown certain efficacy and prospects in the prevention and treatment of GVHD after HSCT, as well as the promotion of implantation and hematopoietic function reconstruction by coinfusion with HSCs. However, most of these studies are from a single center, the number of samples is too small, and many problems remain unclear.

First, the immune regulation of MSCs is affected by immune cells and inflammatory mediators of the body, which is associated with multiple receptors and factors. When there is infection in the body, MSCs play an anti-infection role by promoting the immune response rather than inhibiting the proliferation of T cells [89]. The mechanisms of regulation and differentiation of MSCs in different disease model settings are still unknown.

Second, MSCs are a group of polyclonal cell populations. These clonal subpopulations are different in cell morphology, proliferation potential and cell function. Different cell subpopulations can play different roles, such as immunosuppression, promoting angiogenesis, and promoting hematopoiesis, thus playing a role in different diseases. How to find a better MSC subgroup that can promote hematopoiesis and play an immune regulatory role is still unclear.

Third, factors such as age of patients, type of transplantation, severity of GVHD, source of MSCs, frequency of use of MSCs, and time of use of MSCs may affect the clinical efficacy of MSCs. There is no consensus on the infusion window period, infusion dose, infusion mode and infusion frequency of MSCs for patients after HSCT.

In addition, the long-term safety and efficacy after MSC infusion remain unclear. For example, whether the immunosuppressive effect of MSCs will increase the risk of infection, whether it will increase the risk of secondary tumors after infusion, and whether the biological characteristics and clinical efficacy of MSCs from different tissue sources are consistent have always been unavoidable problems in the clinical use of MSCs.

## Future perspectives

Looking into the future, with the improvement of new technologies such as in vitro induced differentiation, isolation and purification, and gene modification, MSCs with stronger specificity, higher purity and better homing efficiency will be applied to clinical work. Further research on the mechanism of MSCs and searching for better MSC subsets to promote hematopoietic development will be of great significance for the clinical prophylaxis and treatment of GVHD after allo-HSCT. In the future application of MSCs, it should be more important to select the appropriate source of MSCs and formulate appropriate application solutions for different individuals and patients at different stages. Therefore, MSCs, with their unique advantages, hold great promise in the field of hematopoietic stem cell transplantation in the future.

## Data Availability

Not applicable.

## Abbreviations

HSCT: Hematopoietic stem cell transplantation
GVHD: Graft-versus-host disease
MSCs: Mesenchymal stem cells
UCB: Umbilical cord blood
BM-MSCs: MSCs derived from bone marrow
PMSCs: Placenta-derived MSCs
HSCs: Hematopoietic stem cells
UCB-MSCs: MSCs derived from umbilical cord blood
AT-MSCs: Adipose tissue-derived mesenchymal stem cells
allo-HSCT: Allogeneic HSCT
SAA: Severe aplastic anemia
Haplo-HSCT: Haploidentical HSCT
HCT: Hematopoietic cell transplantation
UCBT: Umbilical cord blood transplantation
CBT: Cord blood transplantation
aGVHD: Acute GVHD
cGVHD: Chronic GVHD
NK cells: Natural killer cells
OS: Overall survival
AML: Acute myelogenous leukemia
NML: Non-Hodgkin lymphoma
MM: Multiple myeloma
PFS: Progression-free survival
PMF: Primary myelofibrosis
PNH: Paroxysmal nocturnal hemoglobinuria
